# A New Perspective on Intercalated Disc Organization: Implications for Heart Disease

**DOI:** 10.1155/2010/207835

**Published:** 2010-05-05

**Authors:** Jifen Li, Glenn L. Radice

**Affiliations:** Department of Medicine, Center for Translational Medicine, Jefferson Medical College, Philadelphia, PA 19107, USA

## Abstract

Adherens junctions and desmosomes are intercellular adhesive junctions and essential for the morphogenesis, differentiation, and maintenance of tissues that are subjected to high mechanical stress, including heart and skin. The different junction complexes are organized at the termini of the cardiomyocyte called the intercalated disc. Disruption of adhesive integrity via mutations in genes encoding desmosomal proteins causes an inherited heart disease, arrhythmogenic right ventricular cardiomyopathy (ARVC). Besides plakoglobin, which is shared by adherens junctions and desmosomes, other desmosomal components, desmoglein-2, desmocollin-2, plakophilin-2, and desmoplakin are also present in ultrastructurally defined fascia adherens junctions of heart muscle, but not other tissues. This mixed-type of junctional structure is termed hybrid adhering junction or area composita. Desmosomal plakophilin-2 directly interacts with adherens junction protein alphaT-catenin, providing a new molecular link between the cadherin-catenin complex and desmosome. The area composita only exists in the cardiac intercalated disc of mammalian species suggesting that it evolved to strengthen mechanical coupling in the heart of higher vertebrates. The cross-talk among different junctions and their implication in the pathogenesis of ARVC are discussed in this review.

Arrhythmogenic right ventricular cardiomyopathy (ARVC) is an inherited heart muscle disease estimated to affect approximately 1 in 5,000 individuals [1]. The prominent features are myocytes loss, fibro-fatty tissue replacement, and life-threatening ventricular arrhythmias [2–4]. Approximately a third of patients with ARVC have one or more mutations in genes encoding cardiac desmosomal proteins; hence ARVC is referred to as “a disease of the desmosome” [5, 6]. 

Desmosomes and adherens junctions are intercellular adhesive junctions that anchor intermediate filaments and actin cytoskeleton, respectively, at the plasma membrane of adjoining cells, thereby provide mechanical attachment between the cells, and support the structural and functional integrity of the tissues. Desmosomes consist of three families of proteins, desmosomal cadherins, armadillo proteins, and plakins (Table 1) [7]. Desmosomal cadherins, desmogleins (DSGs), and desmocollins (DSCs) form the extracellular connections by homophilic and heterophilic binding. The cytoplasmic tails of desmosomal cadherins bind to the armadillo protein plakoglobin (PG) and plakophilins (PKP), which in turn bind to the plakin protein, desmoplakin (DP). Desmoplakin links desmosomes to intermediate filament protein. Different desmosomal proteins are differentially expressed in a tissue-specific as well as differentiation-dependent manner. Unlike DSG-1, -3, and -4 and DSC-1 and 3, which are predominantly expressed in the skin epidermis, DSG-2 and DSC-2 are highly expressed in the myocardium of the heart. PKP-1 and PKP-3 are the major epidermal PKPs, whereas PKP-2 is the sole PKP present in cardiac tissue. Desmosomal proteins DP, PG, and plectin are shared by epidermis and myocardium (Table 1). Consistent with differential expression pattern of desmosomal components between heart and skin, human mutations causing ARVC in the genes encoding PG, DP, and DSC-2 are associated with the cardiocutaneous syndrome [8]. In addition to ARVC, patients exhibit woolly hair and palmoplantar keratoderma, and they may also have skin blistering. 

The classical cadherins are calcium-dependent cell adhesion receptors, located in the adherens junction. The extracellular domain of cadherins interacts homophilically mediating strong cell-cell adhesion and plays a key role in the maintenance of tissue structure. In the classic model for adherens junction, the cytoplasmic tail of cadherin interacts in a mutually exclusive manner with either *β*-catenin or PG. *β*-catenin or PG links cadherins to *α*-catenin, and *α*-catenin interacts with the actin cytoskeleton. Gap junction is another intercellular junction in the cell responsible for cell-cell communication and electrical coupling by mediating small molecules and ion transfer between the cells. Each gap junction is composed of two hexameric structures called connexons or hemichannels that dock across the extracellular space and form a permeable pore. Each connexon consists of six transmembrane proteins called connexin (Cx). Cx43 is the most abundant connexin isotype in the heart. The different junctional complexes must be properly organized in the intercalated disc (ICD) of the myocardium to preserve normal mechanical and electrical function of the heart.

Plakoglobin, also known as *γ*-catenin, is the only linker protein present in both desmosomes and adherens junctions in skin and heart [9]. Plakoglobin was also the first component of the desmosome to be implicated in the pathogenesis of ARVC. Studies of individuals from the Greek island of Naxos identified an autosomal recessive inherited ARVC with palmoplantar keratoderma and woolly hair. Gene sequencing revealed a homozygous 2 bp deletion (2157-2158delGT) in the junction plakoglobin gene (*JUP*) in affected individuals [10]. A study of a German family recently reported the first dominantly inherited *JUP* mutation (S39_K40insS) to cause ARVC without cutaneous abnormalities [11]. Importantly, reduced immunoreactive signal of PG at the ICD is a consistent feature in patients with ARVC making it an important diagnostic tool for ARVC in affected individuals [12]. Most recently, studies on cardiac restricted deletion of PG in adult mice have shown similar features to ARVC, including myocytes loss, inflammation, fibrosis, and cardiac dysfunction [13]. 

The mixing of junctional components in the heart was first described in PG null mice. The homozygous PG null animals die between embryonic days 12–16 due to ventricular rupture and hemorrhaging into the pericardial cavity. Desmosomes are not detected in the mutant hearts. Instead extended adherens junctions develop, which contain desmosomal proteins such as desmoplakin, forming “mixed type” adhering junction [14, 15]. In the heart of PKP-2 null embryos, the desmosome-like structures are also not present, and two morphotypes of desmosomes and adherens junctions are found difficult to distinguish [16]. These observations in mutant mice suggest that the formation of hybrid adhering junctions may be compensatory response to weakened adhesion due to loss of desmosomal proteins PG or PKP-2 in the myocardium.

The typical morphological appearance of cardiac intercalated disc at the ultra structural level in the mice is submembranous plaques with electron dense material adjacent to intercellular space between the myocytes (Figure 1(A)). A relatively large junction that anchors primarily bundles of actin myofilaments is fascia adhaerens-like junction, and junction that anchors primarily intermediate filaments is a desmosomal-like junction (Figure 1(A)). For desmosomes, distinct electron dense material is often observed in the intercellular space. During embryonic development, the shape of the individual cardiomyocyte changes from more polygonal to more elongate with alignment of the myofibrils to the longitudinal axis of the cell. Accompanying the morphological change, the junctional components distribute from all round the cell to the sites of myofibril attachment and eventually restrict to the cell-cell contact of polarized adult myocytes (i.e., ICD) [17]. 

By comprehensive immunoelectron microscopy with immunogold DP antibody labeling, Franke et al. observed that in normal heart muscle, DP is located in all plaques of both the desmosome-like and fascia adhaerens-type junctions. Very intensely labeled junctions with DP are seen in the more desmosome-like junctions (Figure 1(B)), whereas equal label intensity of continuous DP is seen in mixed-type junctions or hybrid adhering junctions (Figure 1(C)). Using various antibodies to desmosomal plaque proteins, Franke et al. further found that other desmosomal molecules, PG, PKP-2, DSC-2, and DSG-2, are also not restricted to the desmosome-like junctions but also can be detected in adherens junction structures. This large plaque-coated hybrid structure therefore has been termed an “area composita” [18] (Figure 2). By light and electron microscopy, the molecules known as typical components of fascia adhaerens, including N-cadherin, *α*-catenin, and *β*-catenin, also have been shown to colocalize with desmosomal proteins in the majority of the area composita junctions [19]. These studies suggest that the area composita is an unusual high molecular complexity and the elements that exist in this hybrid structure are intimately associated [19]. Consistent with late maturation of the ICD structure during development, the formation of the extended area composita junction is also a late, primarily postnatal process in mammalian heart [20]. By contrast, in nonmammalian species (fishes, amphibia, birds), adherens junctions and desmosomes remain separate and distinct structures in these adult hearts, suggesting that the formation of the area composita is not only a relatively late process in mammalian ontogenesis but also in vertebrate evolution [20, 21]. 

*α*T-catenin is a recently identified member of *α*-catenin family with restricted expression in testis, brain, and cardiac muscle [22]. Extensive studies from Goossens et al. provided molecular evidence that *α*T-catenin, a unique molecule of adherens junction, functions as cytoskeletal linker protein that specifically brings the desmosomes and adherens junctions together in the intercalated disc of the heart [23]. Using yeast two-hybrid and co-immunoprecipitation, *α*T-catenin was shown to interact specifically with desmosomal PKP-2 implicating a novel molecular linkage between the adherens junction and desmosome. By double *α*T-catenin/PKP-2 immunolabeling electron microscopy, *α*T-catenin is observed to colocalize with other molecules of cadherin/catenin complex, *β*-catenin, and N-cadherin at the fascia adhaerens-like junctions, but also with desmosomal proteins such as PKP-2, DSG-2, and DP. By contrast, no localization of either *α*E-catenin or *β*-catenin could be seen at desmosome-like junctions of the ICD. Based on the biochemical and morphological studies, *α*T-catenin is thought to recruit desmosomal proteins to hybrid adhering junctions, forming a mixed-type, reinforced junction at the ICD that is attached to both the intermediate filaments and actin cytoskeletons (Figure 2). Accordingly, interfering with *α*T-catenin function may impaire intercellular coupling between *α*T-catenin, PKP-2, and the cytoskeleton, which may subsequently result in destabilization of the ICD structure, cardiac dysfunction, as well as cardiac arrhythmia [23]. *α*T-catenin is coexpressed with a closely related family member, *α*E-catenin, in the heart. Most importantly, *α*E-catenin lacks the PKP-2 binding domain; therefore mutations in *α*T-catenin are predicted to have an adverse affect on the organization of the area composita since *α*E-catenin is not able to interact with PKP-2. Human *α*T-catenin gene *CTNN3* has been mapped to chromosome 10q21, a region that links to autosomal dominant familial dilated cardiomyopathy (DCM) [24]. Although genetic screening has not detected any DCM-linked *CTNN3* mutations to date, *α*T-catenin is considered a candidate gene and may be the potential cause of DCM or ARVC [24]. 

It is estimated that as many as 70% of the desmosomal mutations linked to familial ARVC are in the gene coding for PKP-2 [25]. Abnormal expression of gap junction protein Cx43 has been observed in heterozygous human PKP-2 mutations [26, 27]. Similar observations have also been made in patients with mutations in plakoglobin (Naxos disease) or desmoplakin (Carvajal syndrome) genes [5, 28–30]. These studies suggest that abnormalities in intercellular adhesion caused by mutations in desmosomal proteins may promote remodeling of gap junctions, which, in turn, alters cardiac conduction and potentially leads to ventricular arrhythmogenic phenotype in this disease [5]. This hypothesis has been supported in a cellular model in which PKP-2 was knockdown by shRNA [31]. Loss of PKP-2 expression in the cells leads to a decrease in total Cx43 content, a significant redistribution of Cx43 to the intercellular space, and a decrease in dye coupling between cells. GST-pulldown assays have shown that PKP-2 and Cx43 coexist in the same macromolecular complex. Recently, siRNA-mediated reduction of PKP-2 has been shown to disintegrate area composita junction structures in cardiac myocytes [32]. These results provide a possibility that PKP-2 is directly involved in the stabilization of Cx43 within the gap junction plaques [31].

The presence of stable mechanical coupling mediated by N-cadherin/catenin adhesion complex is of paramount importance for maintaining the structural integrity of the heart. We demonstrated that cardiac-specific deletion of N-cadherin in mice (N-cad CKO) causes dissolution of the adherens junction, desmosomes, and area composita resulting in absence of ICD structure in the N-cadherin mutant heart [33]. Gap junction protein Cx43 is also markedly decreased from the ICD in the N-cad CKO mice, leading to spontaneous ventricular tachycardia and sudden cardiac death [34]. In contrast, induced deletion of Cx43 in the adult heart did not affect the structure of the ICD with respect to the spatial organization of adherens junction and desmosome [35]. To our knowledge, N-cad CKO is the first animal model with such a dramatic structural phenotype affecting all the junctional complexes in the heart. Our studies demonstrated that the integrity of ICD structure including the area composita is dependent on N-cadherin function in the adult myocardium [33]. 

In conclusion, the idea that anchoring junctions of the ICD are segregated into distinct domains performing independent functions needs to be reevaluated based on recent data demonstrating novel protein interactions between components from different junction complexes; for example, *α*T-catenin/PKP-2 and PKP-2/Cx43. The different junctional complexes must be properly localized in the ICD to mediate normal mechanical and electrical coupling between cardiomyocytes. The mixed-type, reinforced junction at the ICD may have evolved to maintain the exceptionally large gap junction plaques found in the mammalian heart, which is under high mechanical stress. Given the large number of PKP-2 mutations identified in ARVC patients, it will be imperative to understand the function of this plakophilin in maintaining electrical synchrony in the heart. Recent studies from human genetics and animal models suggest cross-talk between intercellular junctions constituting the ICD, including adherens junction, desmosome, area composita, and gap junctions. Loss-of-function studies in mice have provided important insight into the hierarchical relationship between the different junction complexes; however subtle mutations in ICD proteins will be necessary to understand the molecular mechanisms underlying ARVC. In the future, it will be interesting to know how the area composita is affected in ARVC patients and its implication in the pathogenesis of the disease.

## Figures and Tables

**Figure 1:**
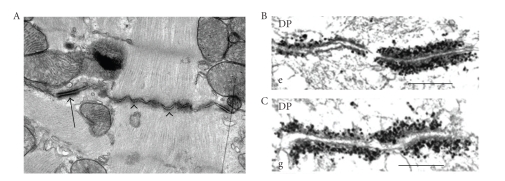
(A) Transmission electron microscopy of intercalated disc in the normal mouse heart, showing the plasma membranes of the adjacent cardiomyocytes with a region structurally resembling a desmosome (arrow) anchoring intermediate filaments, and fascia adhaerens-like structure anchoring predominantly bundles of actin filaments (arrowheads). (B) and (C) Immunoelectron microscopy of the myocardium of mouse heart, showing desmoplakin (DP) antibody labeling with silver amplification at cell-cell junctions. Note that DP immunogold label is enriched in plaques of the junctions. Higher label intensity of DP is desmosome-like structure (the right hand junction in Figure 1(B)), and the fascia adhaerens-like junction is shown in less intensity of DP labeling (the left hand junction in Figure 1(B)). A continuous and equal intensity of DP labeling shows the hybrid junctions (Figure 1(C)) [16]. Figures 1(B) and 1(C) was originally published in “The Journal of Cell Biology, Grossman et al., 2004. doi:10.1083/jcb.200402096” [16].

**Figure 2:**
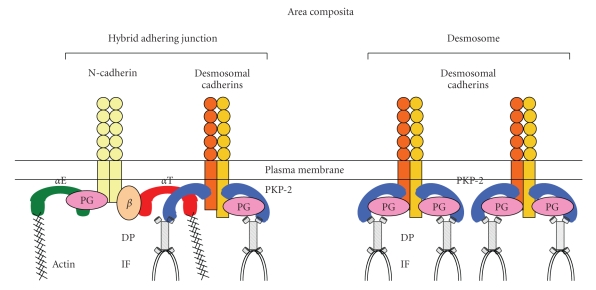
Model for cadherin-based area composita in the heart. *α*T-catenin recruits desmosomal protein, plakophilin- (PKP-) 2 to hybrid adhering junction (left drawing), thereby forming, together with desmosome (right drawing), an area composita, which is an enforced, mixed-type junctional structure attached to both the actin cytoskeleton and the intermediate filament (PG: plakoglobin; DP: desmoplakin).

**Table 1:** The different composition of desmosomes in skin and heart.

	Skin	Heart
Desmosomal cadherins	Desmoglein (DSG) 1–4	Desmoglein (DSG)-2
	Desmocollin (DSC) 1–3	Desmocollin (DSC)-2

Armadillo proteins	Plakoglobin (PG)	Plakoglobin (PG)
	Plakophilin (PKP) 1–3	Plakophilin (PKP)-2

Plakins	Desmoplakin (DP)	
	Plectin	Desmoplakin (DP)
	Envoplakin	Plectin
	Periplakin	

## Abbreviations

ARVC: Arrhythmogenic right ventricular cardiomyopathy
DCM: Dilated cardiomyopathy
ICD: Intercalated disc
DSG: Desmoglein
DSC: Desmocollin
PG: Plakoglobin
PKP: Plakophilin
DP: Desmoplakin
Connexin: Cx.
